# Role of modern immunotherapy in gastrointestinal malignancies: a review of current clinical progress

**DOI:** 10.1186/s13045-017-0454-7

**Published:** 2017-04-24

**Authors:** Zin W. Myint, Gaurav Goel

**Affiliations:** Division of Medical Oncology, Department of Medicine, Markey Cancer Center, University of Kentucky, 800 Rose Street, CC449, Lexington, KY 40503 USA

**Keywords:** Immunotherapy, Immune checkpoint blockade, PD-1, PD-L1, CTLA-4, Gastrointestinal cancers, Esophageal cancer, Gastric cancer, Colorectal cancer, Pancreatic cancer, Hepatocellular carcinoma

## Abstract

Gastrointestinal (GI) cancers are a group of highly aggressive malignancies with a huge disease burden worldwide. There is clearly a significant unmet need for new drugs and therapies to further improve the treatment outcomes of GI malignancies. Immunotherapy is a novel treatment strategy that is emerging as an effective and promising treatment option against several types of cancers. CTLA-4 and PD-1 are critical immune checkpoint molecules that negatively regulate T cell activation via distinct mechanisms. Immune checkpoint blockade with antibodies directed against these pathways has already shown clinical efficacy that has led to their FDA approval in the treatment of several solid tumors including melanoma, non-small cell lung cancer, renal cell carcinoma, urothelial carcinoma, and head and neck cancer. This review will summarize the current clinical progress of modern immunotherapy in the field of GI tumors, with a special focus on immune checkpoint blockade.

## Background

Gastrointestinal (GI) cancers are a group of highly aggressive malignancies with a huge disease burden worldwide. Despite the progress made in the field of cancer therapeutics, there is clearly a significant unmet need for new drugs and therapies to improve upon the efficacy of available anticancer agents and reduce the toxicity of current regimens, with an overall goal of improving the treatment outcomes of GI malignancies.

Immunotherapy is a novel treatment strategy that is emerging as an effective and promising treatment option against several types of cancers. Sir William Coley was the first to use a bacterial immunotoxin to treat a patient with neck sarcoma in 1898 [1]. More recently during the last decade, immunotherapy has shown encouraging results in various tumor types. This progress is largely attributed to identification of new immune-based targets, coupled with our enhanced understanding of tumor immunology and the tumor microenvironment [2]. Cytotoxic T lymphocyte protein-4 (CTLA-4) and programmed cell death protein-1 (PD-1) are critical immune checkpoint molecules that negatively regulate T cell activation via distinct mechanisms. CTLA-4 is a co-inhibitory molecule expressed on activated T cells and T regulatory (Treg) cells. Interaction of the CTLA-4 receptor on T cells with its B7-1/B7-2 ligands on antigen-presenting cells (APCs) inhibits the CD28-mediated T cell stimulatory signal [3]. The inhibition of this receptor-ligand interaction using an anti-CTLA-4 antibody results in the reactivation and proliferation of T cells and also decreases the immunosuppressive Treg cells in the tumor microenvironment [4]. The anti-CTLA-4 antibody ipilimumab is currently approved for the treatment of melanoma. PD-1 is a co-inhibitory receptor expressed on the surface of activated T cells, Treg cells, and monocytes. It interacts with its two ligands, PD-L1 and PD-L2, to provide an inhibitory signal in T cell activation, leading to downregulation of cellular and humoral immune responses [5–7]. Increasing evidence suggests that targeting the PD-1/PD-L pathway is an effective therapeutic strategy to enhance the antitumor immune response. Antibody-mediated blockage of PD-1 or PD-L1 results in the inhibition of this checkpoint, leading to T cell activation and enhanced antitumor activity [8, 9]. PD-1 pathway inhibitors are currently approved by the Food and Drug Administration (FDA) for the treatment of several solid tumors including melanoma, non-small cell lung cancer (NSCLC), renal cell carcinoma (RCC), urothelial carcinoma, and head and neck cancer. Additional potentially targetable checkpoints such as OX40, TIM3, and LAG3 are also being evaluated in ongoing preclinical and clinical studies.

This review will summarize the current clinical progress of modern immunotherapy in the treatment of various GI malignancies, with a special focus on immune checkpoint blockade.

## Esophageal and gastric cancers

Cancers of the upper GI tract are a group of highly aggressive malignancies. In the USA alone, 16,910 new cases of esophageal cancer and 26,370 cases of stomach cancer are estimated to be diagnosed in 2016, and approximately 15,690 and 10,730 deaths will be attributed to these diseases, respectively [10]. It is now well recognized that there exists an urgent unmet need to identify effective novel strategies for the treatment of these patients that are aimed at improving the clinical outcomes beyond the activity of the existing approaches. Targeting the immune checkpoint pathways to activate the host immune system against cancer cells is one such novel approach that is rapidly evolving in the recent years.

A comprehensive molecular characterization of gastric adenocarcinoma as part of The Cancer Genome Atlas (TCGA) project has identified four major genomic subtype of gastric cancer, namely, EBV-positive tumors, microsatellite instability (MSI) tumors, genomically stable tumors, and chromosomally unstable tumors [11, 12]. In the EBV subgroup, there was amplification of *JAK2*, *CD274*, and *PDCD1LG2* [11]. *CD274* and *PDCD1LG2* encode PD-L1 and PD-L2, respectively, which suggests a potential role of PD-1/PD-L1 pathway inhibitors in the treatment of gastric cancer.

High expression of PD-L1 is associated with a poor prognosis in esophageal cancers. Ohigashi et al. studied the role of PD-L1 and PD-L2 expression in 41 human esophageal cancer resection specimens and demonstrated that expression of either PD-L1 or PD-L2 is a significant prognostic marker in patients with squamous cell carcinoma of the esophagus [13]. A significant positive correlation between mRNA and protein expression was observed for both PD-L1 and PD-L2. In this study cohort, overall survival (OS) for PD-L1- or PD-L2-positive patients was significantly worse than those absent for these markers (*P* = 0.025 and *P* = 0.003, respectively). In multivariate analysis, the PD-L1 and PD-L2 status continued to remain a significant independent prognostic factor (*P* = 0.0001). This led to the hypothesis that PD-L status may be a critical factor to promote tumor growth and metastasis in esophageal cancer. In another study involving 101 distal esophageal adenocarcinoma primary resection specimens without preoperative chemotherapy or radiotherapy, Loos et al. demonstrated that high tumor PD-L1 expression was significantly associated with poor survival (HR = 2.92; *P* < 0.001) [14]. The prognostic significance of PD-L1 expression persisted even in multivariate analysis after adjusting for the TNM stage and tumor grade. A similar prognostic role of PD-L1 has been observed in gastric cancer as well. Zheng et al. performed a retrospective analysis using 80 advanced gastric cancer patients and 40 healthy controls, to evaluate the association between circulating PD-L1 expression and prognosis [15]. Circulating PD-L1 expression was tested by ELISA and was found to be significantly upregulated in gastric cancer patients compared to healthy controls. In the study by Wu et al., it was shown that normal gastric tissues do not express PD-L1, but it was detected in 42% of the gastric cancer tissues [16]. The level of PD-L1 expression by immunohistochemistry (IHC) significantly correlated with tumor size, lymph node metastases, and patient survival. Saito et al. investigated the PD-1 expression on CD4^+^ and CD8^+^ T cells and its relationship with immune evasion in gastric cancer patients [17]. The PD-1 expression on T cells from gastric cancer patients was significantly higher than normal controls and was found to be related to disease progression. The above data provides sufficient evidence to suggest that the PD-1/PD-L pathway plays a critical role in the evasion of antitumor host immune responses in esophageal and gastric cancers. Accordingly, developing therapies aimed at targeting this immune checkpoint pathway is a potentially promising strategy that can lead to improvement in the treatment outcomes for this devastating disease.

The multi-cohort, phase Ib KEYNOTE-012 trial was one of the early studies to evaluate the safety and efficacy of anti-PD-1 antibody pembrolizumab in previously treated, PD-L1-positive, advanced solid tumors (NCT01848834) [18]. The tumors were defined as PD-L1-positive if the marker was present on ≥1% tumor cells or if any positive staining was present in the stroma. This gastric cancer cohort enrolled 39 patients with PD-L1-positive recurrent or metastatic adenocarcinoma of the stomach or gastroesophageal junction (GEJ). An overall response rate (ORR) of 22% (8 PR (partial response)) was reported in 36 evaluable advanced gastric cancer patients. Pembrolizumab demonstrated a manageable safety profile with grade 3–4 treatment-related adverse events (TRAEs) observed in 13% patients. In this study, no association was observed between response and higher PD-L1 expression on tumor cells, as assessed with the clinical trial assay. In another similar multi-cohort phase Ib trial, single-agent pembrolizumab was evaluated in PD-L1-positive advanced solid tumors (KEYNOTE-028, NCT02054806). The esophageal carcinoma cohort enrolled 23 patients with squamous cell carcinoma (SqCC; 74%) or adenocarcinoma (22%) of the esophagus or GEJ, which had progressed on the standard therapies [19]. The primary endpoint of ORR was estimated at 30% in this cohort of heavily pretreated patients. At the time of data cutoff in November 2015, four out of seven responses were still ongoing and the median duration of response (DoR) had not been reached. Single-agent pembrolizumab exhibited a manageable toxicity profile with 17% of the patients experiencing grade 3 TRAEs. The encouraging clinical activity observed in these studies formed the basis for larger phase II and III studies of anti-PD-1 therapies in gastric, esophageal, and GEJ cancers.

KEYNOTE-059 is an ongoing phase II multi-cohort study in recurrent or metastatic gastric or GEJ adenocarcinoma patients (NCT02335411). The three study cohorts in this trial include pembrolizumab plus fluoropyrimidine and cisplatin in treatment-naïve patients, pembrolizumab monotherapy in previously treated patients, and pembrolizumab alone in previously untreated patients. The preliminary safety data from the pembrolizumab plus 5-fluorouracil (5-FU) and cisplatin combination cohort were presented at the 2016 American Society of Clinical Oncology (ASCO) annual meeting [20]. At the time of data cutoff, 18 patients had been treated with this combination regimen. Grade 3–4 TRAEs were noted in 67% patients, none of which were attributed to pembrolizumab. Another ongoing phase II trial is evaluating pembrolizumab as a monotherapy in patients with previously treated advanced adenocarcinoma or SqCC of the esophagus or GEJ (KEYNOTE-180, NCT02559687). Exploratory analyses in this study will include evaluation of the immune-related gene expression profiles and PD-L1 expression status as predictors of pembrolizumab efficacy.

Multiple phase III studies involving anti-PD-1 therapy in gastric and esophageal cancers are currently in progress. KEYNOTE-061 is a randomized, open-label study designed to compare the efficacy and safety of pembrolizumab versus standard-of-care paclitaxel in the second-line treatment of metastatic or unresectable gastric or GEJ adenocarcinoma (NCT02370498). KEYNOTE-062 is another randomized phase III study containing three arms which will compare the safety and efficacy of pembrolizumab monotherapy, versus 5-FU plus cisplatin, versus all three agents combined together in the first-line treatment of PD-L1-positive advanced gastric or GEJ adenocarcinoma (NCT02494583). Another phase III trial (KEYNOTE-181) will enroll approximately 600 patients to compare the efficacy of pembrolizumab relative to single-agent chemotherapy (paclitaxel, docetaxel, or irinotecan) in patients with previously treated advanced adenocarcinoma or SqCC of the esophagus or GEJ (NCT02564263).

Avelumab is a fully human anti-PD-L1 IgG1 antibody. The safety and clinical activity of avelumab as a first-line maintenance or second-line therapy in patients with advanced gastric or GEJ cancer were evaluated in the phase Ib JAVELIN trial (NCT01772004) [21]. Of the 151 patients who have been treated with avelumab in this study so far, grade ≥3 TRAEs were reported in 10% patients. There was one treatment-related death secondary to hepatic failure/autoimmune hepatitis. The unconfirmed ORR was 10% (6 PR) in the second-line therapy arm and 9% (2 CR, 6 PR) in the first-line maintenance therapy arm. The disease control rate (DCR) was 29 and 57% and median progression-free survival (PFS) was 6 and 12 weeks in the two treatment arms, respectively. Encouraged by the acceptable safety profile and promising clinical activity of single-agent avelumab, two large randomized phase III trials of avelumab in gastric cancer have been initiated (NCT02625623, NCT02625610).

Immune checkpoint inhibition with anti-CTLA-4 antibody has also been explored in the treatment of upper GI tract cancers. Single-agent ipilimumab was evaluated in a randomized phase II trial of previously treated patients with locally advanced or metastatic gastric and GEJ cancers (NCT01585987) [22]. The study was terminated early after the interim analysis due to the lack of demonstrable clinical activity with ipilimumab. Combining anti-CTLA-4 and anti-PD-1 together is another potential immunotherapeutic strategy that is being explored in the ongoing CheckMate-032 trial (NCT01928394). This phase I/II, open-label study is evaluating the efficacy of nivolumab alone or in combination with ipilimumab, in chemotherapy-refractory advanced solid tumors. The three treatment arms in this study include nivolumab 3 mg/kg, nivolumab 1 mg/kg plus ipilimumab 3 mg/kg, and nivolumab 3 mg/kg plus ipilimumab 1 mg/kg. The preliminary results from the gastric cancer cohort (*n* = 160) demonstrated an ORR of 14, 26, and 10% in the three treatment arms, respectively [23]. The grade 3–4 TRAEs occurred in 17, 45, and 27% of patients, respectively.

Table 1 provides a summary of the selected ongoing immunotherapy clinical trials in various GI malignancies.

**Table 1:** Summary of selected ongoing immunotherapy clinical trials in gastrointestinal malignancies

NCI identifier	Study phase	Study agent	Mode of action of immunotherapy agent
Esophageal cancer			
NCT02642809	0	Pembrolizumab + radiotherapy	Anti-PD-1 Ab
NCT02735239	I/II	Durvalumab + chemotherapy ± radiotherapy	Anti-PD-L1 Ab
NCT02559687 (KEYNOTE-180)	II	Pembrolizumab	Anti-PD-1 Ab
NCT02644863, NCT01691625	II	Chemotherapy ± autologous dendritic cells and cytokine-induced killer cells (DC-CIK)	Adoptive cellular therapy
NCT02564263 (KEYNOTE-181)	III	Pembrolizumab vs. chemotherapy	Anti-PD-1 Ab
Gastric cancer			
NCT02443324	I	Pembrolizumab + ramucirumab	Anti-PD-1 Ab, anti-VEGFR2 Ab
NCT02310464	I	OBI-833	Cancer vaccine
NCT02689284	Ib/II	Pembrolizumab + margetuximab	Anti-PD-1 Ab, anti-HER2-neu Ab
NCT02340975	Ib/II	Tremelimumab vs. durvalumab vs. tremelimumab + durvalumab	Anti-CTLA-4 Ab, anti-PD-L1 Ab
NCT02317471	I/II	Autologous gp96 vaccination ± chemotherapy	Heat shock protein purified from autologous tumor cells
NCT02617134	I/II	Anti-MUC1 CAR T cells	Adoptive cellular therapy
NCT02862561, NCT02873520	I/II	Precision cell immunotherapy ± chemotherapy	Dendritic cell suspension
NCT01783951	I/II	DC-CIK + S-1	Adoptive cellular therapy
NCT02632201	I/II	Pluripotent killer T cells expressing Ab for HER-2 (PIK-HER2)	Adoptive cellular therapy
NCT02370498 (KEYNOTE-061)	III	Pembrolizumab vs. paclitaxel	Anti-PD-1 Ab
NCT02494583 (KEYNOTE-062)	III	Pembrolizumab vs. pembrolizumab/cisplatin/5-FU vs. placebo/cisplatin/5-FU	Anti-PD-1 Ab
NCT02625610 (JAVELIN Gastric 100)	III	Avelumab vs. chemotherapy	Anti-PD-L1 Ab
NCT02625623 (JAVELIN Gastric 300)	III	Avelumab + BSC vs. chemotherapy + BSC	Anti-PD-L1 Ab
Colorectal cancer			
NCT02512172	I	Pembrolizumab + romidepsin and/or 5-azacitidine	Anti-PD-1 Ab
NCT02856425	I	Pembrolizumab + nintedanib	Anti-PD-1 Ab
NCT02777710	I	Durvalumab + CSF-1R TKI	Anti-PD-L1 Ab
NCT02754856	I	Durvalumab + tremelimumab	Anti-PD-L1 Ab, anti-CTLA-4 Ab
NCT02559024	I	MEDI6469	Anti-OX40 Ab
NCT01890213	I	AVX701	CEA-based cancer vaccine
NCT02617134	I/II	Anti-MUC1 CAR T cells	Adoptive cellular therapy
NCT02900664	Ib	PDR001 + either CJM112 or EGF816 or canakinumab or trametinib	Anti-PD-1 Ab
NCT02460198 (KEYNOTE-164)	II	Pembrolizumab	Anti-PD-1 Ab
NCT02860546	II	Nivolumab + TAS-102	Anti-PD-1 Ab
NCT02466906	II	RhGM-CSF	Recombinant human GM-CSF
NCT02448173	III	OncoVAX + surgery	Cancer vaccine
NCT02563002 (KEYNOTE-177)	III	Pembrolizumab vs. chemotherapy	Anti-PD-1 Ab
NCT02788279	III	Atezolizumab ± cobimetinib vs. regorafenib	Anti-PD-L1
NCT02280278	III	CIK	Cytokine-induced killer cells
Pancreatic cancer			
NCT02734160	I	Durvalumab + galunisertib	Anti-PD-L1 Ab
NCT02529579	I/II	Gemcitabine ± iAPA-DC/CTL	Adoptive cellular therapy
NCT02718859	I/II	NK cells + irreversible electroporation	Natural killer cells
NCT02305186	I/II	Chemoradiation ± pembrolizumab	Anti-PD-1 Ab
NCT01781520	I/II	DC-CIK + S-1	Adoptive cellular therapy
NCT01896869	II	FOLFIRINOX + ipilimumab + allogeneic GM-CSF vaccine	Anti-CTLA-4 Ab, vaccine
NCT02648282	II	Cyclophosphamide + pembrolizumab + GVAX + SBRT	Anti-PD-1 Ab, vaccine
NCT02558894	II	Durvalumab ± tremelimumab	Anti-PD-L1 Ab, anti-CTLA-4 Ab
NCT02243371	II	GVAX + CRS-207 ± nivolumab	Vaccine, anti-PD-1 Ab
Hepatocellular carcinoma			
NCT02843802	I/II	NK cells + cryosurgery	Natural killer cells
NCT02873442	I/II	Precision cells + TACE	Dendritic cell suspension
NCT02239900	I/II	Ipilimumab + SBRT	Anti-CTLA-4 Ab
NCT01658878	I/II	Nivolumab; nivolumab + ipilimumab	Anti-PD-1 Ab, anti-CTLA-4 Ab
NCT02839954	I/II	CAR-pNK cell	Adoptive cellular therapy
NCT02632006	I/II	PIK-PD-1 cells	Adoptive cellular therapy
NCT02715362	I/II	GPC3-CAR T cells	Adoptive cellular therapy
NCT02702414	II	Pembrolizumab	Anti-PD-1 Ab
NCT02519348	II	Durvalumab + tremelimumab vs. durvalumab vs. tremelimumab	Anti-PD-L1 Ab, anti-CTLA-4 Ab
NCT02487017	II	TACE ± DC-CIK	Adoptive cellular therapy
NCT02256514	II	Hepcortespenlisimut-L	Cancer vaccine
NCT01174121	II	Autologous tumor infiltrating lymphocytes (TILs)	Adoptive cellular therapy
NCT02562755	III	Sorafenib ± Pexa-Vec	Vaccinia virus-based cancer vaccine
NCT02576509	III	Nivolumab vs. sorafenib	Anti-PD-1 Ab
NCT02702401 (KEYNOTE-240)	III	Pembrolizumab vs. BSC	Anti-PD-1 Ab
NCT02232490	III	Hepcortespenlisimut-L vs. placebo	Cancer vaccine

## Colorectal cancer

Colorectal cancer (CRC) is a major public health problem in the USA and globally. In the USA, it is the second leading cause of cancer mortality, and in 2016, it is estimated that nearly 50,000 deaths will be attributed to this disease [10]. Within the last decade itself, several new drugs (regorafenib, ramucirumab, TAS-102) have been approved by the FDA for the treatment of CRC, but the magnitude of clinical benefit with these agents has continued to remain modest [24–28]. Consequently, novel treatment strategies such as immunotherapy are being evaluated to further improve the outcomes of this disease.

Almost a decade ago, Galon and colleagues demonstrated that the type, density, and distribution of immune cells inside colorectal tumors can predict the clinical outcome more accurately than the conventional TNM staging [29]. In this study, the numbers of total T cells (CD3^+^), cytotoxic effector T cells (CD8^+^), and memory T cells (CD45RO^+^) were evaluated in the tumor core and at the invasive margin. The investigators demonstrated that tumors with high levels of CD3^+^ T cells in the core as well as at the invasive margin were associated with the best clinical outcome, independent of the TNM stage. In a subsequent study, the prognostic role of tumor-infiltrating lymphocytes (TILs) was also demonstrated in metastatic CRC lesions [30]. In this study, high TIL density in the metastatic sites conferred a greater response to chemotherapy treatment and was associated with a longer PFS. In an effort to promote quantification of the immune infiltrate inside the tumor, the “Immunoscore” methodology has been defined [31]. Recently, in a worldwide consortium-based analysis of 1,336 stage I/II/III colon cancer patients, the standardized immunoscore assay was validated as a prognostic marker, and a “high” immunoscore was found to be associated with longer time-to-tumor recurrence [32].

It is now well established that two distinct immunologic subtypes of CRC exist, according to the mismatch repair (MMR) status, namely, MMR-proficient and MMR-deficient. Microsatellite instability (MSI) in colorectal tumors occurs either due to a germline mutation in one of the four DNA MMR genes (*MLH1*, *MSH2*, *MSH6*, *PMS2*) or from epigenetic silencing of *MLH1* caused by promoter region hypermethylation. This results in multiple frameshift and missense mutations of DNA coding sequences, which produce a large number of aberrant proteins that are recognized as “non-self” antigens and trigger an antitumor immune response [33]. In fact, the overall number of frameshift mutations in a microsatellite-unstable colorectal tumor has been shown to correlate with TIL density [34]. Llosa et al. analyzed the genetics and immune microenvironment of colorectal tumors in parallel and demonstrated that tumors with high T cell infiltrate had defects in MMR resulting in MSI [35]. MSI-Hi CRCs were shown to upregulate expression of several immune checkpoints (PD-1, PD-L1, CTLA-4, LAG-3, IDO) in the TILs, stroma, or tumor invasive front compartments, enabling the tumor cells to survive. The MSI-Hi subtype of CRC thus represents an inherently sensitive population for immunotherapy-based treatment approaches.

In the first-in-human (FIH) phase I trial of nivolumab that enrolled treatment-refractory solid tumors, only one patient showed a durable complete response (CR), and this was a patient with MSI-Hi CRC [36]. The other 19 CRC patients did not have any tumor response, and all of these had microsatellite-stable (MSS) disease. A phase II study evaluated the clinical activity of pembrolizumab monotherapy in patients with previously treated, progressive metastatic tumors, with and without MMR deficiency (NCT01876511) [37]. The patients were enrolled in three cohorts, MMR-deficient CRC, MMR-proficient CRC, and MMR-deficient non-colorectal tumor cohorts. In this study, the MMR-deficient CRCs were reported to have encouraging responses to pembrolizumab. The updated results of 53 patients, including 28 patients in the MMR-deficient CRC cohort and 25 patients in the MMR-proficient CRC cohort, were presented at the 2016 ASCO annual meeting [38]. The ORR and DCR were 50 and 89% for MMR-deficient CRC and 0 and 16% for MMR-proficient CRC, respectively. The median PFS was not reached for MMR-deficient CRC and was 2.4 months for MMR-proficient CRC (HR = 0.135; 95% CI, 0.043 to 0.191; *P* = 0.0001). The median OS was not reached in the MMR-deficient CRC cohort, as opposed to 6 months in the MMR-proficient CRC cohort (HR = 0.247; 95% CI, 0.117 to 0.589; *P* = 0.001). These results suggest that MMR-deficient CRC tumors receive durable benefit from anti-PD-1 therapy. Based on these encouraging results, a phase II study (KEYNOTE-164, NCT02460198) and a phase III study (KEYNOTE-177, NCT02563002) of pembrolizumab in MMR-deficient advanced CRC are ongoing to confirm these early observations. MSI-Hi tumors are known to be associated with high neo-antigenic burden; consequently, the tumor mutational burden is currently being evaluated as a biomarker for PD-1/PD-L1 therapeutic response in CRC [39].

Dual immune checkpoint inhibition with a combination of anti-CTLA-4 and anti-PD-1 antibodies was examined in a phase II study (CheckMate-142) that evaluated nivolumab with or without ipilimumab in metastatic CRC (NCT02060188). The interim results demonstrate that the combination of nivolumab plus ipilimumab is associated with encouraging clinical activity especially in the MMR-deficient subgroup (4-month PFS rate of 80%, 5-month OS rate of 100%) [40].

MMR deficiency occurs in less than 5% of patients with metastatic CRC [41]. Despite the compelling responses observed in the MMR-deficient tumors, treatment with immune checkpoint blockade has been largely disappointing for the vast majority of advanced CRC patients with MMR-proficient disease [8, 37, 38, 42]. This is unlike gastric cancer, where response to immune checkpoint blockade in MMR-proficient disease has been observed in a few instances [43]. Ongoing research is focused on understanding why MMR-proficient CRC remains largely refractory to immune checkpoint blockade. An important goal is to develop strategies aimed at improving the sensitivity of MMR-proficient CRC towards immunotherapy, as they comprise the vast majority of colorectal tumors. A potential strategy is to combine immune checkpoint inhibitors with additional agents/therapies that have immunomodulatory properties, to stimulate the immune response in the MMR-proficient tumors. Several ongoing clinical trials are actively testing this hypothesis.

A phase II study is evaluating the safety and abscopal effect of pembrolizumab when administered in combination with radiotherapy or tumor ablation, in patients with MMR-proficient metastatic CRC (NCT02437071) [44]. Out of 11 patients in the pembrolizumab plus radiotherapy cohort, one patient demonstrated PR in the non-irradiated lesion. No responses were observed among the eight evaluable patients in the pembrolizumab plus ablation cohort.

In preclinical models, MAPK/ERK kinase (MEK) inhibition is associated with upregulation of major histocompatibility complex (MHC)-1 on cancer cells and infiltration of T cells in tumors, leading to enhanced anti-PD-L1 activity. This formed the basis of a phase Ib study that combined a MEK inhibitor, cobimetinib, with an anti-PD-L1 antibody, atezolizumab, in patients with advanced solid tumors (NCT01988896).The preliminary results from 23 CRC patients (22 KRAS mutant, 1 wild type) who have been treated with this combination showed that four patients (3 MMR-proficient, 1 unknown) achieved a PR and another five had SD [45]. These promising results have led to the design of a large phase III study which is evaluating the combination of cobimetinib plus atezolizumab in MMR-proficient advanced CRCs (NCT02788279).

Epigenetic priming using inhibition of histone deacetylases (HDAC) and DNA methyltransferases (DNMT) can induce susceptibility to immune checkpoint therapy in preclinical models. This is being evaluated in an ongoing pilot study that combines 5-azacitidine (DNMT inhibitor) or romidepsin (HDAC inhibitor) or both with the PD-1 inhibitor pembrolizumab in MMR-proficient advanced CRC (NCT02512172).

Upregulation of immunosuppressive cofactor PD-L1 is an important mechanism by which tumor cells can escape host T cell immunity. Emerging evidence now suggests that chemotherapeutic agents can regulate PD-L1 expression on cancer cells, which may have an impact on antitumor immunity and immune evasion [46–48]. Moreover, many chemotherapeutic agents including taxanes, gemcitabine, doxorubicin, 5-azacitidine and 5-FU are known to have immunostimulatory effects in addition to their conventional cytotoxic activity [49, 50]. For example, 5-FU facilitates antigen expression by tumor cells, antigen uptake by dendritic cells, and subsequent cross-penetration of tumor antigens [51]. Therefore, combining chemotherapy with an immune checkpoint inhibitor that can counteract the effects of PD-L1 is another potential therapeutic strategy that is being explored in clinical trials (NCT01633970, NCT02375672, NCT02291289).

## Pancreatic cancer

Pancreatic ductal adenocarcinoma (PDAC) is a biologically aggressive disease with a dismal prognosis and limited therapeutic options. It is the fourth leading cause of cancer-related death in the USA for both men and women [10]. In 2016 alone, approximately 53,000 new cases of pancreatic cancer are estimated to be diagnosed in the USA, and approximately 42,000 patients will die from their disease. It is now well established that pancreatic cancer carcinogenesis is driven by alteration in multiple genes that regulate oncogenic pathways in the tumor cells and the neighboring microenvironment [52, 53]. A prominent histological hallmark of PDAC is desmoplastic reaction, which results in the formation of dense stroma in the tumor microenvironment [54]. This not only presents as a mechanical barrier to immune cells and the effective delivery of anticancer agents to the tumor cells but also contributes to the development of an antiangiogenic, hypoxic, and immunosuppressed tumor microenvironment. Despite our improved understanding of the involved molecular pathways, there has been no clinically meaningful improvement in the 5-year survival rates for patients with pancreatic cancer. Consequently, many novel treatment strategies against pancreatic cancer are currently in preclinical and clinical development [53].

Several cancer vaccines aimed at stimulating the host immune system by generating humoral and/or cellular immune responses have also been evaluated in the treatment of pancreatic cancer. However, this strategy has not demonstrated a meaningful clinical benefit in any phase III clinical trial till date. Algenpantucel-L is a whole-cell allogeneic pancreatic cancer vaccine composed of two irradiated human pancreatic cell lines that have been engineered to overexpress murine enzyme alpha(1,3)-galactosyltransferase. In a phase II adjuvant trial of resected pancreatic cancer, algenpantucel-L demonstrated encouraging activity when combined with radiation, 5-FU, and gemcitabine [55]. This led to a large phase III adjuvant trial in pancreatic cancer that compared gemcitabine with or without algenpantucel-L followed by chemoradiation (IMPRESS, NCT01072981). A total of 722 pancreatic cancer patients who had undergone surgical resection were enrolled in the study. The study did not meet its primary endpoint; the median OS was 30.4 and 27.3 months in the control and study groups, respectively. Another phase III trial is examining the role of neoadjuvant FOLFIRINOX or gemcitabine/nab-paclitaxel with or without algenpantucel-L, followed by chemoradiation in borderline resectable or locally advanced unresectable pancreatic cancer patients (PILLAR, NCT01836432). The study has completed its planned enrollment of over 300 patients in December 2015.

GV1001 is a telomerase peptide vaccine shown to prolong survival when combined with granulocyte-macrophage colony-stimulating factor (GM-CSF) in a phase I/II study of unresectable pancreatic cancer patients [56]. However, the phase III study comparing gemcitabine with or without GV1001 in advanced unresectable pancreatic cancer was terminated early due to lack of survival benefit in the GV1001 arm (NCT00358566). Another phase III trial has examined the efficacy of GV1001 in combination with gemcitabine plus capecitabine (TeloVac, ISRCTN4382138) [57]. The median OS was not statistically significantly different in the chemotherapy group than in the chemo-immunotherapy group.

GVAX is composed of allogeneic pancreatic cancer cells that are genetically modified to secrete GM-CSF. CRS-207 is a live-attenuated *Listeria monocytogenes*-based vaccine that can induce listeriolysin O- and mesothelin-specific T cell responses. The combination of GVAX/CRS-207 was shown to extend survival in metastatic pancreatic cancer patients, in a phase II study [58]. Subsequently, the combination of GVAX/CRS-207 was evaluated in a larger phase IIb clinical trial (ECLIPSE) of pretreated advanced pancreatic cancer patients (NCT02004262). In this open-label, three-arm trial, 303 patients with metastatic pancreatic cancer were randomized to receive either GVAX/CRS-207 combination, CRS-207 alone, or chemotherapy. This study also failed to meet its primary endpoint, with the median OS of 3.8, 5.4, and 4.6 months in the three study arms, respectively.

Wilms’ tumor gene (*WT1*) is overexpressed in several malignancies including PDAC. The efficacy of *WT1* peptide vaccine plus gemcitabine was examined in a randomized phase II study of locally advanced or advanced pancreatic cancer patients [59]. In the subgroup of patients with metastatic disease, the combination of *WT1* plus gemcitabine was associated with a superior PFS, as compared to gemcitabine alone (133 versus 76 days; HR = 0.48; 95% CI, 0.30–0.77; *P* = 0.008). IMM-101 is heat-killed *Mycobacterium obuense*, which is capable of modulating the innate and adaptive immune systems in response to cancer [60]. IMAGE-1 was a randomized (2:1) phase II study designed to explore the safety and tolerability of IMM-101 in combination with gemcitabine versus gemcitabine alone, as first-line treatment in advanced pancreatic cancer (NCT01303172) [61]. The recently reported results confirmed that IMM-101 plus gemcitabine was as safe and well tolerated as gemcitabine alone. Moreover, in the predefined metastatic subgroup, OS was significantly improved in favor of IMM-101 plus gemcitabine combination (7.0 versus 4.4 months; HR = 0.54; 95% CI, 0.33–0.87; *P* = 0.01). It would be interesting to see whether *WT1* peptide vaccine and IMM-101 continue to retain their clinical efficacy when tested in large phase III trials.

As in other malignancies, the immunotherapeutic strategy of targeting the immune checkpoints such as CTLA-4 and PD-1/PD-L1 is also being evaluated in the treatment of pancreatic cancer. However, immune checkpoint blockade by itself has shown limited clinical responses in pancreatic cancer. For example, a phase II trial of single-agent ipilimumab failed to demonstrate any significant antitumor activity in advanced pancreatic cancer [62]. This is largely attributed to the lack of intratumoral effector T cells and an immunologically quiescent microenvironment of pancreatic cancer. Due to the lack of meaningful clinical benefit of individual immunotherapeutic agents against pancreatic cancer, a strategy of combining immunotherapy with additional therapies is being actively investigated. The combination of ipilimumab plus gemcitabine was evaluated in a phase Ib dose-finding study of advanced pancreatic cancer (NCT01473940). Dose escalation was performed in a standard 3 + 3 fashion. The maximum tolerated dose (MTD) was established at gemcitabine 1000 mg/m^2^ plus ipilimumab 3 mg/kg dose. Out of 16 patients enrolled in the study, 2 patients experienced PR and 5 had SD. The median PFS and OS were 2.5 and 8.5 months, respectively [63]. Checkpoint inhibition is also being combined with pancreatic cancer vaccines with a goal to improve the T cell-specific responses. An ongoing phase II study is comparing combination of GVAX/CRS-207 with or without nivolumab in patients with metastatic pancreatic cancer who have failed one prior chemotherapy regimen for metastatic disease (STELLAR, NCT02243371). In another pilot study, nivolumab is being combined with dendritic cell vaccine in metastatic pancreatic cancer. In seven patients who have been treated till date, two PR have been observed so far [64].

Combined blockade of immune checkpoints is also being explored in pancreatic cancer. An ongoing phase II study is evaluating durvalumab with or without tremelimumab in previously treated metastatic pancreatic cancer patients (ALPS, NCT02558894). Combining anti-PD-1 therapy with targeted agents is another therapeutic strategy. An open-label, randomized phase II study compared the efficacy of a Bruton’s tyrosine kinase (BTK) inhibitor, acalabrutinib, with or without pembrolizumab in patients with metastatic pancreatic cancer (NCT02362048) [65]. The combination was noted to have encouraging preliminary activity and an acceptable side effect profile. In the combination arm, 3 PR and 5 SD were reported among the 23 patients who were evaluable for response.

## Hepatocellular carcinoma

Hepatocellular carcinoma (HCC) is the fifth most common cancer worldwide and the second leading cause of cancer-related deaths in men [66]. Due to its insidious onset and rapid progression, most cases are detected at an advanced stage. Infection with hepatitis B virus (HBV) and hepatitis C virus (HCV), chronic alcoholism, and fatty liver disease are the major risk factors for HCC. Recent evidence suggests that the phenomenon of epithelial-mesenchymal transition (EMT) plays a central role in the pathogenesis of HCC and is also likely responsible for the development of metastases, tumor heterogeneity, and therapeutic resistance [67]. Currently, sorafenib, a multi-targeted tyrosine kinase inhibitor (TKI), is the only systemic agent approved for the treatment of advanced HCC, but the treatment is usually associated with significant toxicity and confers only a modest survival benefit [68]. Moreover, no therapeutic agents are currently approved for sorafenib-resistant disease. Therefore, there is an urgent unmet need for breakthrough in systemic treatment of advanced HCC.

Immune checkpoint inhibitors, including anti-CTLA-4 and anti-PD-1 antibodies, are being evaluated in the treatment of HCC and have demonstrated preliminary evidence of efficacy with a manageable toxicity profile. The antitumor and antiviral effect of the anti-CTLA-4 antibody tremelimumab in patients with HCC and chronic HCV infection was tested in a pilot clinical trial (NCT01008358). The PR rate was 17.6% and the DCR was 76.4% [69]. The time to tumor progression (TTP) was 6.5 months in this study. A significant drop in viral load was also observed with tremelimumab therapy. Tremelimumab has also been combined with subtotal ablation (trans-arterial chemoembolization (TACE), radiofrequency ablation (RFA), or cryoablation) in a study that enrolled HCC and biliary tract cancers (NCT01853618). So far, 41 patients have been enrolled in the study, and 4 (23.5%) confirmed PR were observed among the 17 evaluable patients [70]. The median PFS in the evaluable HCC patients was 5.7 months. Tumor biopsies obtained at 6 weeks demonstrated accumulation of TILs, and there was peripheral blood activation of CD4^+^ and CD8^+^ T cells in the responding patients. Among the 12 patients with underlying HCV infection, 10 patients experienced a marked reduction in viral load with the combination therapy. The findings from this study suggest that tremelimumab combined with subtotal ablation using TACE, RFA, or cryoablation is safe and a feasible strategy in the treatment of advanced HCC.

The anti-PD-1 agent nivolumab is currently being evaluated in a phase I/II study (CheckMate-040) of advanced HCC patients who have previously failed, have refused, or are intolerant of sorafenib (NCT01658878). In the dose escalation (0.1, 0.3, 1, 3, and 10 mg/kg) phase of this study, patients with a Child-Pugh score ≤7 were enrolled in three parallel cohorts based on the underlying disease etiology: no active hepatitis virus infection, HBV-infected, and HCV-infected [71]. The primary endpoint of the study is safety, and the secondary endpoints include antitumor activity by RECIST and DoR. Out of a total of 51 patients in this phase of the study, TRAEs were observed in 39 patients (77%), with rash (20%) and elevation in aspartate aminotransferase (AST; 20%) being the most common. The most common grade 3–4 TRAEs included elevation of AST (10%), alanine aminotransferase (ALT; 6%), and lipase (6%) levels. The MTD was not reached in any study cohort. Efficacy evaluation in 48 patients demonstrated durable treatment response, and disease stabilization was observed across all dose levels and cohorts. The ORR was determined at 15% (3 CR, 4 PR), and the median DoR was 23.7 months. In the subsequent dose expansion phase of this study, 206 patients with advanced HCC were treated with nivolumab at 3 mg/kg and were enrolled in four cohorts (uninfected sorafenib-naïve/intolerant, uninfected sorafenib progressors, HBV-infected, and HCV-infected) [72]. The grade 3–4 TRAEs were observed in 28 patients (14%); the most common included elevation in AST and ALT (3% each). A decline in tumor burden was observed in 39% patients. Preliminary efficacy results show an ORR of 9% among 91 evaluable patients. The 6-month OS rate was 69% (95% CI, 0.43–0.85). Responses were observed independent of the tumor PD-L1 expression. Based on the encouraging preliminary data from the CheckMate-040 trial, a randomized phase III study comparing the efficacy of nivolumab versus sorafenib in advanced HCC was designed and is currently ongoing (CheckMate-459, NCT02576509).

Anticancer vaccines are also being actively tested in the treatment of HCC. For example, Pexa-Vec (pexastimogene devacirepvec) is an oncolytic and immunotherapeutic vaccinia virus that disrupts tumor vasculature and mediates antitumor immunity via GM-CSF expression. In a randomized phase II dose-finding trial, intratumoral injection of Pexa-Vec in advanced HCC patients demonstrated an acceptable safety profile and a significant increase in OS in the high-dose group [73, 74]. A randomized phase III study to compare the efficacy and tolerability of Pexa-Vec followed by sorafenib versus sorafenib alone in patients with advanced HCC is currently ongoing (PHOCUS, NCT02562755). Another oral therapeutic vaccine, hepcortespenlisimut-L, is currently being evaluated in an ongoing phase III clinical trial (NCT02232490) after it demonstrated encouraging activity in the phase II study [75].

## Conclusions

GI malignancies have been traditionally considered to be poorly immunogenic; however, increasing evidence now suggests that these tumors are recognized by the immune system. Immune checkpoint blockade with antibodies targeting B7 immunoglobulin superfamily molecules such as CTLA-4, PD-1, and PD-L1 is showing promising clinical activity in multiple GI tumors including esophageal, gastric, colorectal, and liver cancers. In fact, one of the most significant achievements witnessed in the field of immunotherapy has been the success of PD-1 pathway blockade in MSI-Hi colorectal and non-colorectal tumors [76]. The therapeutic strategy of combining immune checkpoint inhibitors with additional immunostimulatory therapies, targeted agents, chemotherapy drugs, or radiotherapy appears to be a promising approach that might help further unleash the antitumor immunity against several types of GI cancers. However, treatment with such immunostimulatory therapies is not completely devoid of side effects, and cases of potentially fatal immune-related adverse events have been reported [77, 78]. The identification of clinically relevant predictive and prognostic biomarkers will therefore help define subgroups of GI cancer patients who are most likely to benefit from various immunotherapeutic approaches [79]. This is critical for the success of immunotherapy and is therefore the focus of ongoing translational research in the field.
